# A hybrid GA–LP scenario-based framework for day-ahead scheduling of PV–BES systems with profit–robustness trade-off

**DOI:** 10.1038/s41598-026-62641-w

**Published:** 2026-07-20

**Authors:** Ibrahim Alsaidan

**Affiliations:** Department of Electrical Engineering, College of Engineering, Qassim University, Buraydah, Saudi Arabia

**Keywords:** Energy storage, Battery management systems, Solar energy, Optimization, Genetic algorithm, Linear programming, Energy science and technology, Engineering, Mathematics and computing

## Abstract

This paper proposes a scenario-based scheduling framework for a photovoltaic–battery energy storage (PV–BES) system participating in day-ahead energy arbitrage markets. The proposed framework accounts for uncertainties in both PV generation and electricity prices. A hybrid optimization approach is developed, combining a genetic algorithm (GA) with a linear programming (LP) model. The optimization problem is decomposed into two layers. In the first layer, the GA determines the BES commitment schedule, while in the second layer, a coupled LP is solved to obtain the shared BES dispatch schedule, held fixed across all scenarios, with the grid exchange power varying per scenario. A robustness weight is incorporated to control the trade-off between profitability and robustness. Solving the problem across multiple values of robustness weight yields a Pareto frontier, enabling operators to select a scheduling strategy aligned with their risk preference. A case study based on real PV generation and electricity price data is conducted to validate the effectiveness of the proposed framework. The results are further compared with those obtained from three benchmark approaches: deterministic MILP, a naive rule-based method, and classical robust optimization.

## Introduction

Battery energy storage systems are increasingly being integrated across a wide range of energy systems and market structures, including day-ahead and real-time energy markets, reserve and ancillary service markets, microgrid applications, distribution network management, and virtual power plant operations^1–5^. In particular, the integration of BES with photovoltaic plants has expanded the opportunities to enhance profitability in day-ahead energy markets. This is due to the ability of BES to be charged during low-price periods and discharged during high-price periods in a process known as energy arbitrage.

In day-ahead energy markets, participants are required to submit binding bids one day ahead of real-time operation^6^. For a PV–BES system, these bids are composed of the BES commitment schedule, which specifies whether the BES is charging, discharging, or idle, and the corresponding dispatched schedule at each time interval. Any deviation from the submitted day-ahead schedule during real-time operation may result in imbalance charges, which are typically settled at real-time prices that can differ from the day-ahead clearing price due to the realization of uncertain conditions^7,8^.

The main challenge in day-ahead scheduling stems from the fact that the economic performance of the submitted bids is affected by uncertain parameters that are only realized during real-time operation. Therefore, a scheduling framework that accounts for these uncertainties is required to ensure reliable economic performance. In the PV–BES system considered in this work, uncertainty arises from two sources: PV generation, which depends on climatic conditions, and electricity prices, which are influenced by complex interactions among market participants and network conditions. In such situations, existing forecasting methods cannot guarantee a level of accuracy that market participants can rely on.

Since uncertainty representation is central to day-ahead PV–BES scheduling, existing scheduling approaches can be categorized based on how uncertainty is represented, the level of solution conservatism, and the required data. These approaches can be grouped into deterministic, stochastic, and robust optimization methods^9^. Deterministic approaches overlook uncertainty and rely solely on the day-ahead forecast to solve the scheduling problem. While they are computationally tractable and straightforward to implement, they often perform poorly during real-time operation, potentially resulting in reduced revenue and constraint violations. This limitation is discussed by Vejdan and Grijalva^10^, whose work presents a linear optimization model that aims to maximize energy arbitrage profit in day-ahead and real-time markets. Their results demonstrate that failing to consider uncertainties in the day-ahead market reduces the achievable revenue in practice. Despite this limitation, deterministic approaches remain widely used in the literature^1,2,11^.

To address the limitations of deterministic scheduling, stochastic optimization methods represent uncertainty using probability distributions or probability-weighted scenario realizations. The objective of such methods is typically to find a solution that performs well on average across multiple possible realizations of the uncertain parameters. A common formulation is the two-stage stochastic optimization structure, where day-ahead commitment decisions are made in the first stage, while recourse actions are taken in the second stage after the uncertain parameters are realized. This structure has been applied to energy storage and PV–BES scheduling problems^3,12,13^, demonstrating improved performance compared with deterministic approaches.

For example, García-González et al.^12^ applied a two-stage optimization approach to the day-ahead scheduling problem of a co-located wind farm and pumped storage plant, considering uncertainty in both electricity prices and wind generation. Ren and Guan^13^ introduced a two-stage stochastic model for BES day-ahead scheduling under different market execution conditions. Despite their ability to represent uncertainty explicitly, two-stage stochastic formulations assume that recourse actions can be taken after uncertainty is realized. In day-ahead electricity markets, however, submitted schedules are binding, and deviations from the accepted schedule may result in imbalance charges. Therefore, recourse-based formulations may not fully reflect the operational requirements of day-ahead market participation. Alternatively, several studies have proposed simplified scenario-based stochastic optimization models that do not include a recourse stage^14–16^. While these models reduce formulation complexity and partially address the limitations of two-stage stochastic optimization, they still rely on reliable probability information or scenario weights, which are difficult to obtain in practice.

To overcome the need for probability information, the third class is based on robust optimization (RO) methods. Unlike stochastic methods, RO does not require probability distributions of the uncertain parameters. Instead, uncertainty is modeled using a continuous uncertainty set, typically represented by interval, polyhedral, or ellipsoidal sets^17–21^. The objective in classical RO is commonly formulated as a min-max problem, aiming to maximize performance under the worst-case realization within the defined uncertainty set. Attarha et al.^19^ proposed an affinely adjustable robust scheduling framework for a PV–BES system, where joint uncertainty in solar generation and electricity prices is characterized using a polyhedral uncertainty set, with the robustness level controlled via a budget of uncertainty. Similarly, Choi et al.^20^ developed a MILP-based robust scheduling framework for a PV–BES system, representing PV generation uncertainty using a box uncertainty set to obtain the optimal BES schedule. While these approaches eliminate the requirement for probability distribution information, they introduce other limitations. Specifically, since RO problems are formulated as min–max problems, duality theory is typically employed to reformulate them into equivalent deterministic counterparts. This transformation increases the number of decision variables and, consequently, the computational burden. Moreover, by optimizing exclusively against the worst-case realization, classical min-max approaches tend to produce overly conservative schedules that sacrifice average performance, particularly when worst-case scenarios are rare or extreme in practice^17^.

Based on the review above, several limitations remain in existing day-ahead scheduling approaches. Many existing studies consider only a single source of uncertainty, either renewable generation or electricity price, but not both simultaneously. Existing scenario-based methods optimize a single expected-profit objective, without providing a mechanism to control the trade-off between average profit and worst-case protection, offering operators no principled basis for selecting a risk-aware schedule. Furthermore, the recourse structures adopted in two-stage stochastic formulations allow commitment decisions to be revised after uncertainty is realized, which does not reflect the binding nature of day-ahead market submissions where deviations from the submitted schedule may result in imbalance charges. Finally, stochastic optimization methods rely on probability distributions to characterize uncertainty, which are often difficult to obtain reliably in practice. The development of a PV–BES scheduling framework that accounts for joint PV generation and price uncertainty, while balancing robustness and profitability, therefore remains an important research gap.

To address these limitations, this work proposes a day-ahead scheduling framework for a PV–BES system. The proposed framework differs from existing scenario-based approaches in three specific respects. First, uncertainty in both PV generation and electricity price is modeled jointly through a set of paired scenarios, capturing the simultaneous effect of both sources on scheduling performance. Second, a robustness weight is incorporated into the optimization objective, enabling operators to explicitly control the trade-off between average profit and worst-case profit across scenarios. Solving the model across the full range of robustness weight values yields a Pareto frontier that supports risk-aware operational decision-making, a capability not provided by the existing scenario-based methods reviewed above. Third, the proposed framework enforces a strict non-anticipative commitment structure in which a single BES commitment schedule is determined before any scenario is realized and held fixed across all scenarios, with only the grid exchange power allowed to vary per scenario. This structure accurately reflects the binding commitment requirements of day-ahead energy markets and eliminates the need for a recourse stage. The framework does not require probability distribution estimates and is validated through case studies based on real PV generation and electricity price data.

## Proposed PV–BES scheduling framework

**Fig. 1:**
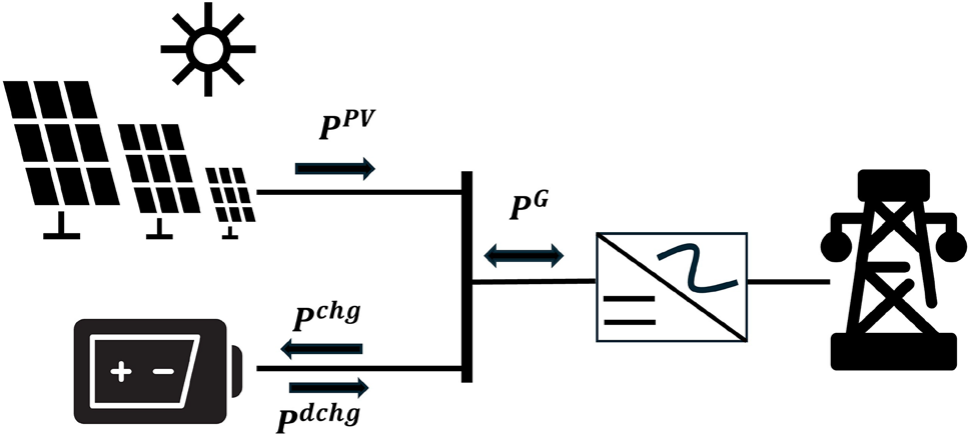
Schematic diagram of the PV–BES grid-connected system.

In this work, the day-ahead scheduling problem is formulated for a utility-scale PV–BES system under joint uncertainty in PV generation and electricity prices. A schematic diagram of the considered system is shown in Fig. 1. The BES can be charged either from the PV system or from the main grid. The PV–BES system is assumed to operate as a price taker, implying that its operation does not influence the market electricity price. This assumption is reasonable when the plant capacity is small relative to the total generation capacity of the grid. The system operator is responsible for managing the power exchange between the PV, BES, and the grid.

**Fig. 2:**
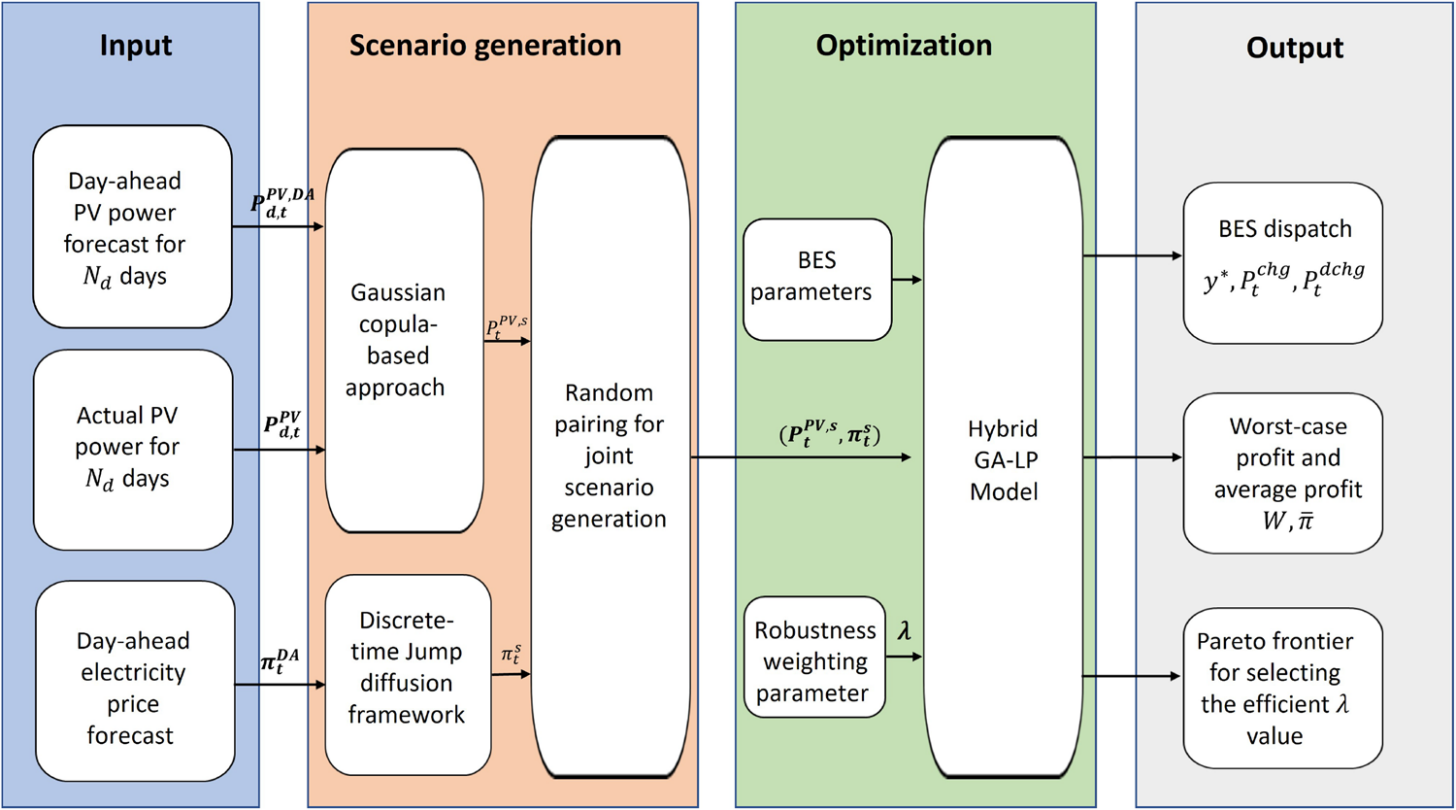
Overview of the main processing steps of the proposed framework.

The proposed framework is based on a two-layer optimization model that enables the PV–BES system operator to evaluate the trade-off between robustness and profitability in day-ahead energy arbitrage. The overall structure of the framework is illustrated in Fig. 2. Day-ahead electricity price forecasts and historical data of forecasted and actual PV generation are provided as inputs to the framework. These data are used to generate scenarios that capture the underlying uncertainties in PV generation and electricity price. Electricity price scenarios are generated using a jump–diffusion model, while PV generation scenarios are constructed using a Gaussian copula-based approach. A set of jointly paired PV–price scenarios is then formed through a random pairing process under the price-taker independence assumption. The hybrid GA–LP optimization model is then applied to determine the optimal day-ahead BES commitment and dispatch schedules across the full range of robustness weight values. To account for uncertainty in PV generation and electricity prices, the proposed framework relies on a set of representative scenarios. The methodology for generating these scenarios is described in the following subsection.

## Electricity price and PV scenario generation

### Electricity price scenarios generation

Different forecasting methods have been used to predict electricity prices in energy markets^22^. These methods are subject to forecasting errors arising from the mismatch between the forecasted and actual values, which can impact the economic return of market participants. To account for this uncertainty, a simplified jump-diffusion model is adopted in this work to generate day-ahead electricity price scenarios.

In financial modeling, Brownian motion is commonly used to describe the continuous evolution of asset prices. However, this approach cannot capture sudden changes or price spikes arising from unexpected market events. To overcome this limitation, jump–diffusion models, such as the Merton model, were introduced to represent discontinuous price movements^23^. The Merton model is expressed by the following stochastic differential equation:

1 $$\begin{aligned} \frac{dS_t}{S_{t^-}} = \left( \mu - \kappa k \right) dt + \sigma \, dW_t + (J - 1) \, dN_t \end{aligned}$$

Equation  1 consists of three terms: a drift term, a diffusion term, and a jump term. The drift term represents the expected long-term growth rate of the price, adjusted for the average impact of jumps. The diffusion term, which is based on Brownian motion, represents the continuous price fluctuations. The jump term captures the unexpected price spikes and is modeled by Poisson process.

A discrete-time approximation of this model is adopted in this work to generate the electricity price scenarios set. The modified equation becomes:

2 $$\begin{aligned} \pi ^{s}_t = \pi ^{DA}_t \times \left( 1 + \sigma Z^s_t + \kappa _t^s \delta ^s_t \right) \qquad \forall t\in T, \forall s\in S \end{aligned}$$

where $\pi ^{DA}_t$ is the day-ahead electricity price forecast at time interval *t*, and $Z_t^s$ is a standard normal random variable representing the price fluctuation. This random noise is controlled using the volatility parameter *σ*. The value of *σ* depends on the market and can be estimated using historical data if available. The spike in the electricity market price that may occur due to unexpected incidents, such as loss of transmission line or generator outage, is simulated in this model using the Bernoulli distribution. That is $\kappa _t^s \sim \text {Bernoulli}(p_{\text {spike}})$ acts as a binary random indicator for spike occurrence: it equals 1 with probability $p_{\text {spike}}$, introducing an electricity price spike with size *δ*, and 0 otherwise. The sets *T* and *S* represent the scheduling time intervals and the generated scenarios, respectively.

**Fig. 3:**
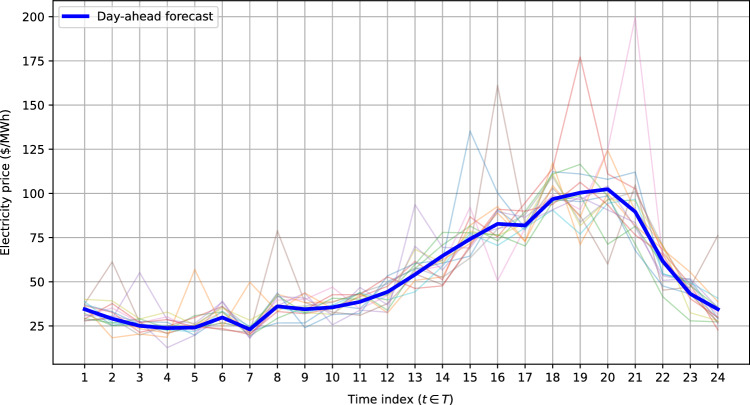
Samples of generated electricity price scenarios with base volatility *(σ ):16* *%*, spike probability ($p_{\text {spike}}$): 4 *%*, and size of the spike (*δ*): *2×*.

This simplified jump diffusion model is sufficient to generate representative price scenarios when applied to the day-ahead electricity price forecast. Figure 3 shows a sample of the generated scenarios using this model based on day-ahead electricity price data retrieved from^24^. The volatility parameter *σ* was set to 16*%* to represent a literature-informed level of day-ahead price uncertainty in the PJM market. Conejo et al.^25^ report that weekly day-ahead price forecasting errors in PJM range from approximately 6*%* for advanced time-series methods to 25*%* during high-volatility summer periods under naive forecasting assumptions, a range further corroborated across multiple electricity markets by O’Connor et al.^22^. The selected value of *σ* = 16*%* lies within this documented range and should therefore be interpreted as a literature-informed scenario-generation parameter reflecting plausible day-ahead price uncertainty faced by market participants when submitting day-ahead bids.

The spike probability $$p_{\text {spike}}$$ was set to 4*%* to represent the occasional occurrence of extreme price deviations in the jump component of the scenario-generation model. Ullrich^26^ analyzed high-frequency electricity spot prices across several wholesale electricity markets, including PJM, and reported PJM spike-frequency estimates of 3.1*%* under the baseline detection lag (i=0) and 7.8*%* under the skip-one lag (i=1). The selected value $$p_{\text {spike}}$$ = 4*%* is therefore close to the lower end of the reported PJM spike-frequency range. It is used as a conservative scenario-generation parameter to introduce rare but possible spike events without making the generated price scenarios overly dominated by extreme jumps. The spike-size multiplier was set to *δ*= 2*×*, which represents a moderate spike magnitude relative to the extreme price behavior reported by Ullrich^26^ where PJM prices reached a maximum of 1020 $/MWh compared with a mean value of 45.37 $/MWh. Accordingly, both $$p_{\text {spike}}$$ and *δ* are treated as conservative scenario-generation parameters rather than direct statistical estimates of hourly spike probability or average historical spike size.

Together, these parameter values provide a literature-informed basis for generating representative price scenarios that reflect day-ahead price uncertainty faced by market participants in the PJM market. The generated scenarios exhibit reasonable variability and occasional price spikes, as illustrated in Fig. 3.

### PV scenario generation

To model the uncertainty in PV generation, a Gaussian copula-based approach is used to generate realistic PV generation scenarios. This method enables preserving both the empirical marginal distributions of forecast errors at each hour and their temporal dependence across the scheduling horizon.

Let $T_s \subseteq T$ denote the set of sun-hour time periods considered for PV scenario generation. For each hour $t \in T_s$, historical PV data are used to construct the distribution of forecast errors. Uncertainty is represented using a multiplicative error model, defined as the ratio of the actual PV output ($P_{d,t}^{PV}$) to the corresponding day-ahead forecast ($P_{d,t}^{PV,DA}$). For historical day *d* and hour *t*, the multiplicative error (*ξ*) is computed as

3 $$\begin{aligned} \xi _{d,t} = \frac{P_{d,t}^{PV}}{\max \!\left( P_{d,t}^{PV,DA}, \varepsilon _{\min }\right) }, \qquad \forall d \in D, \ \forall t \in T_s \end{aligned}$$

where $\varepsilon _{\min }$ is a small positive constant introduced to avoid numerical instability when the forecasted PV output is close to zero, and *D* denotes the set of historical days.

Based on the historical multiplicative errors, the empirical cumulative distribution function (ECDF) is constructed as

4 $$\begin{aligned} \hat{F}_t(x) = \frac{1}{N_d} \sum _{d=1}^{N_d} \textbf{1}\!\left\{ \xi _{d,t}\le x\right\} , \qquad \forall t \in T_s \end{aligned}$$

where $\textbf{1}\{\cdot \}$ denotes the indicator function. The corresponding inverse ECDF is defined as

5 $$\begin{aligned} \hat{F}^{-1}_{t}(u) = \inf \left\{ x \in \mathbb {R} \,:\, \hat{F}_{t}(x) \ge u \right\} , \qquad u \in (0,1), \ \forall t \in T_s . \end{aligned}$$

This formulation preserves the empirical marginal distribution of PV forecast errors at each sun hour.

In addition to marginal distributions, it is essential to capture the temporal dependence of forecast errors across different hours. To this end, an empirical temporal correlation matrix is constructed over the sun-hour horizon. The sample mean and variance of the multiplicative errors at hour *t* are given by

6 $$\begin{aligned} \bar{\xi }_{t} = \frac{1}{N_d} \sum _{d=1}^{N_d} \xi _{d,t}, \qquad \forall t \in T_s \end{aligned}$$

7 $$\begin{aligned} \operatorname {Var}(\xi _t)=\frac{1}{N_d-1}\sum _{d=1}^{N_d} \left( \xi _{d,t} - \bar{\xi }_{t} \right) ^2, \qquad \forall t \in T_s \end{aligned}$$

and the covariance between hours *t* and *k* is computed as

8 $$\begin{aligned} \operatorname {Cov}(\xi _{t}, \xi _{k}) = \frac{1}{N_d - 1} \sum _{d=1}^{N_d} (\xi _{d,t} - \bar{\xi }_{t})(\xi _{d,k} - \bar{\xi }_{k}), \qquad \forall (t,k) \in T_s \times T_s \end{aligned}$$

The corresponding correlation coefficient is

9 $$\begin{aligned} \rho _{t,k} = \frac{\operatorname {Cov}(\xi _t,\xi _k)}{\sqrt{\operatorname {Var}(\xi _t)}\,\sqrt{\operatorname {Var}(\xi _k)}}, \qquad \forall (t,k) \in T_s \times T_s . \end{aligned}$$

By collecting all coefficients, the temporal correlation matrix $\textbf{R}$ is obtained. If necessary, $\textbf{R}$ is adjusted to ensure positive semi-definiteness before applying Cholesky decomposition:

10 $$\begin{aligned} \textbf{R} = \textbf{L}\textbf{L}^\textsf{T}, \end{aligned}$$

where $\textbf{L}$ is a lower triangular matrix.

To generate the PV scenarios, a vector of independent standard normal variables $\textbf{Z}^{(s)} \sim \mathcal {N}(\textbf{0},\textbf{I})$ is sampled for each scenario *s*. Correlated Gaussian samples are then obtained as

11 $$\begin{aligned} \textbf{Y}^{(s)} = \textbf{L}\textbf{Z}^{(s)}, \end{aligned}$$

which follow $\textbf{Y}^{(s)} \sim \mathcal {N}(\textbf{0},\textbf{R})$, preserving the temporal correlation structure defined by $\textbf{R}$. These correlated Gaussian samples are then mapped to uniform marginals using the standard normal CDF:

12 $$\begin{aligned} U_t^{(s)} = \Phi \!\left( Y_t^{(s)} \right) , \qquad \forall s \in S, \ \forall t \in T_s . \end{aligned}$$

The simulated multiplicative error is then obtained as

13 $$\begin{aligned} \xi _t^{(s)} = \hat{F}_t^{-1}\!\left( U_t^{(s)}\right) , \qquad \forall s\in S, \ \forall t \in T_s . \end{aligned}$$

Finally, each PV generation scenario for the target day is reconstructed by applying the sampled multiplicative error to the day-ahead forecast ($P_t^{PV,DA}$) as

14 $$\begin{aligned} P_t^{PV,s}= {\left\{ \begin{array}{ll} 0, & t \notin T_s, \\ \min \!\left\{ \max \!\left( 0,\,P_t^{PV,DA}\,\xi _t^{s}\right) ,\,P^{PV,R}\right\} , & t \in T_s, \end{array}\right. } \qquad \forall s\in S. \end{aligned}$$

In this work, historical PV data from^27^ are used to generate scenarios. A total of 90 days are considered, including 45 days before and 45 days after the target day, to capture variability within the same season. The target day is July 16, 2006, and the dataset spans from June 1 to August 30, 2006. Figure 5 shows examples of the generated PV scenarios. To verify the statistical realism of the generated PV scenarios, a validation study was conducted using the historical PV dataset employed in the scenario generation process. Since the Gaussian copula model generates forecast-error realizations that are subsequently applied to the target-day forecast, the validation was performed using the daily forecast-error ratio, defined as the ratio between actual and forecasted daily PV energy.

15 $$\begin{aligned} r_d = \frac{E^{\textrm{act}}_d}{E^{\textrm{DA}}_d} \qquad \forall d \in D \end{aligned}$$

16 $$\begin{aligned} r_s = \frac{E^{\textrm{scen}}_s}{E^{\textrm{DA}}_{\textrm{target}}} \qquad \forall s \in S \end{aligned}$$

Table 1 summarizes the statistical properties of both distributions. The generated scenarios exhibit close agreement with the historical data in terms of mean, standard deviation, median, and upper percentile values.

**Table 1:** Statistical comparison between historical and generated daily forecast-error ratios.

Metric	Historical	Generated
Mean	0.997	1.012
Standard deviation	0.196	0.175
5th percentile	0.685	0.713
Median	1.009	1.023
95th percentile	1.260	1.264

**Fig. 4:**
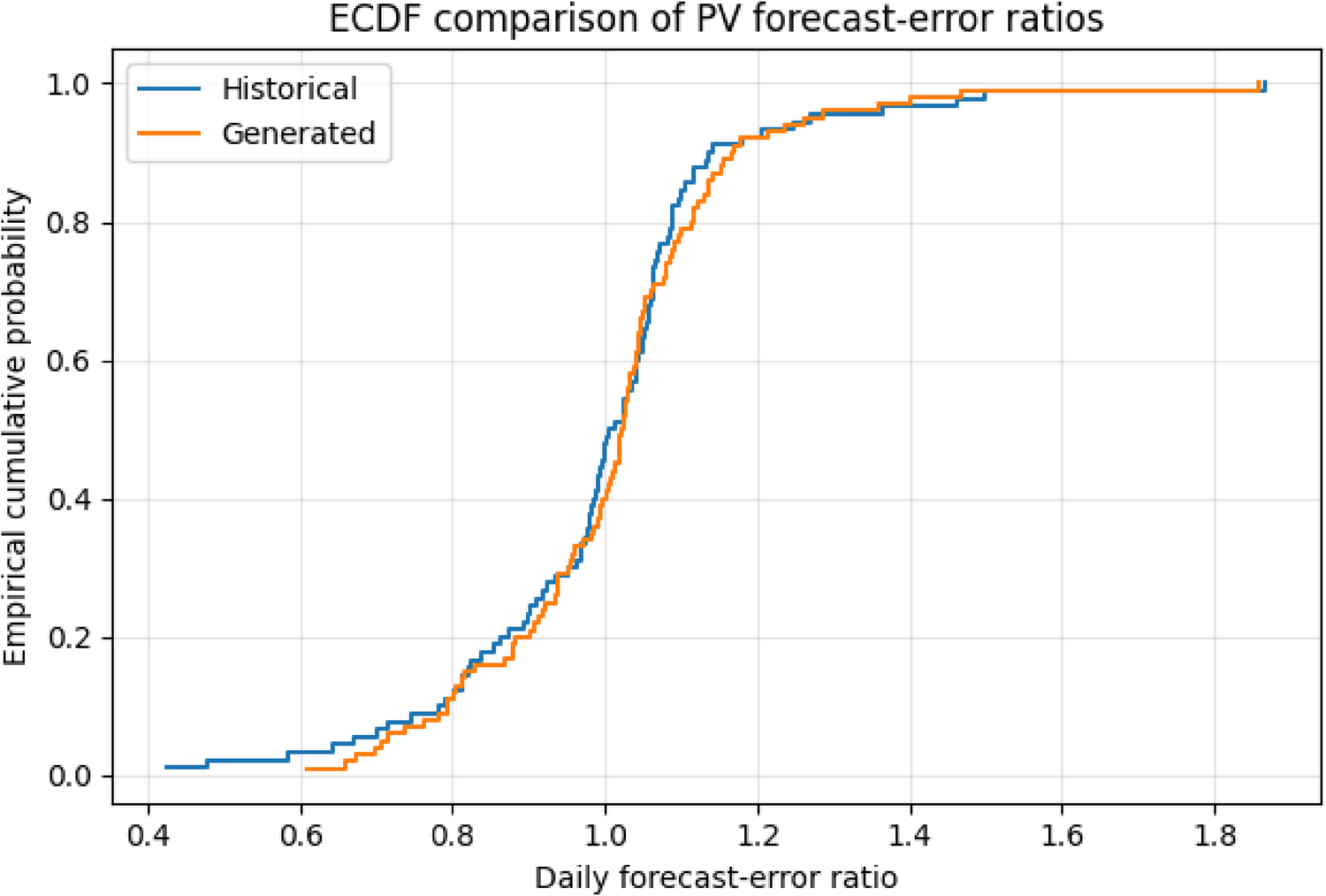
Empirical cumulative distribution function (ECDF) comparison of historical and generated PV daily forecast-error ratios.

**Fig. 5:**
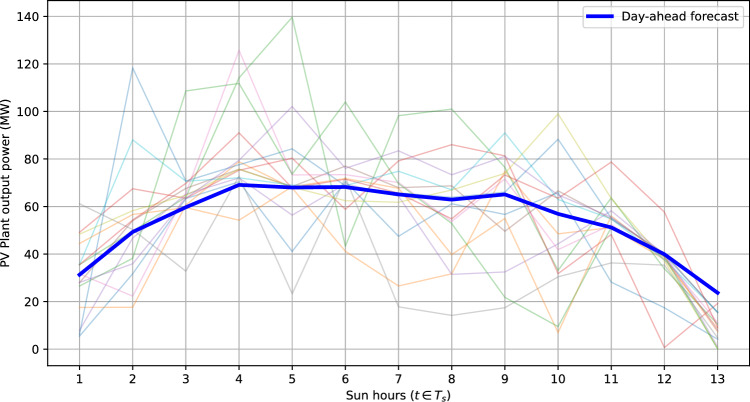
Examples of the generated PV scenarios over the sun-hour scheduling horizon $T_s$.

Figure 4 compares the empirical cumulative distribution functions (ECDFs) of the historical and generated forecast-error ratios. The close overlap between the two distributions indicates that the generated scenarios successfully reproduce the historical forecast-error behavior. This observation is further supported by a Kolmogorov–-Smirnov test, which yielded a p-value of 0.893, indicating no statistically significant difference between the historical and generated distributions (Fig. 5).

### Random pairing joint scenarios

The PV–BES system is treated as a price taker, meaning that its operation does not influence market-clearing prices. Under this assumption, the residual uncertainty in PV generation, represented by the multiplicative forecast errors described in the previous subsection, is primarily driven by local weather and irradiance conditions. The residual uncertainty in electricity prices, represented by multiplicative deviations around the day-ahead price forecast using the jump-diffusion model, reflects broader market dynamics. Therefore, the two residual uncertainties are assumed to be statistically independent. It is noted that this assumption applies to the residual uncertainties around the day-ahead forecasts rather than to the absolute PV generation and electricity price values. Any systematic seasonal or hourly relationship between PV generation and electricity prices is already reflected in the corresponding day-ahead forecasts and is therefore not part of the uncertainty being modelled.

Accordingly, the generated PV and electricity price scenarios are combined into joint (PV, price) pairs using random pairing with a fixed seed, yielding $N_s = 100$ joint scenarios. This approach preserves the marginal distributions of both uncertainty sources while accurately reflecting their statistical independence. The fixed seed ensures full reproducibility of the generated scenario set.

## Hybrid GA-LP model

The paired PV–electricity price scenarios are passed to the hybrid GA-LP model together with the BES parameters and the robustness weight range. The model determines the optimal day-ahead BES commitment schedule, which defines the operating mode, and the BES dispatch schedule, which defines the charge/discharge power at each hour. The robustness weight (*λ*) allows the operator to control the degree of protection against the worst-case scenario. By solving the model across the full range of *λ* values, a Pareto frontier is generated that enables the operator to identify the the preferred trade-off between robustness and profitability.

The first layer employs GA to determine the BES commitment schedule (i.e., charge, discharge, or idle) at each hour, represented by the decision variable $y_t$. GA is well known for solving discrete decision variable optimisation problems, making it appropriate choice for this layer. Although GA is selected in this work, other metaheuristic methods such as particle swarm optimisation (PSO), simulated annealing (SA), and Grey Wolf Optimiser (GWO) can also be applied within the same framework.

The BES commitment schedule is passed to the second layer, which solves a coupled LP problem across all scenarios to determine the optimal charge/discharge power ($P_t^{\textrm{chg}}, P_t^{\textrm{dchg}}$), and state of charge ($SOC_t$). The BES dispatch schedule is held fixed across all scenarios, while the grid exchange power varies according to the realized price and PV generation in each scenario. The proposed structure reflects the practical constraints of day-ahead energy markets. This eliminates the need to revise the day-ahead schedule during real-time operation, avoiding potential imbalance charges and economic losses. The following subsections present the mathematical formulation of each layer in detail.

### First layer: genetic algorithm

Metaheuristic methods have been widely used in scheduling and combinatorial optimization problems, particularly when the solution space is large and the decision variables are discrete^28,29^. In this work, a GA is adopted to search for the BES commitment schedule, which specifies the BES operating mode at each hour. The commitment schedule is represented by a ternary decision variable ($y_t$), defined as follows:

- $y_t = +1$: BES is scheduled to discharge at hour *t*
- $y_t = 0$: BES is idle at hour *t*
- $y_t = -1$: BES is scheduled to charge at hour *t*

Accordingly, the complete BES commitment schedule is represented by the chromosome vector:

17 $$\begin{aligned} y = [y_1, y_2, \dots , y_{24}] \in {[-1, 0, +1]}^{24} \end{aligned}$$

As a result, the search space contains $3^{24} \approx 2.82 \times 10^{11}$ possible commitment schedules, which makes conventional optimization methods impractical and motivates the use of a metaheuristic approach such as GA.

For a given commitment schedule $\boldsymbol{y}$ and robustness weight *λ*, the fitness function is defined as the objective value of the coupled LP solved in the second layer. Since the second layer is formulated as an LP, the optimal solution is guaranteed to be globally optimal. Consequently, the fitness value returned to the GA represents the exact maximum achievable weighted profit for the given commitment schedule across all $N_s$ scenarios. This structure ensures that the GA efficiently explores the discrete search space while the LP layer guarantees optimality of the continuous dispatch for each candidate schedule.

**Fig. 6:**
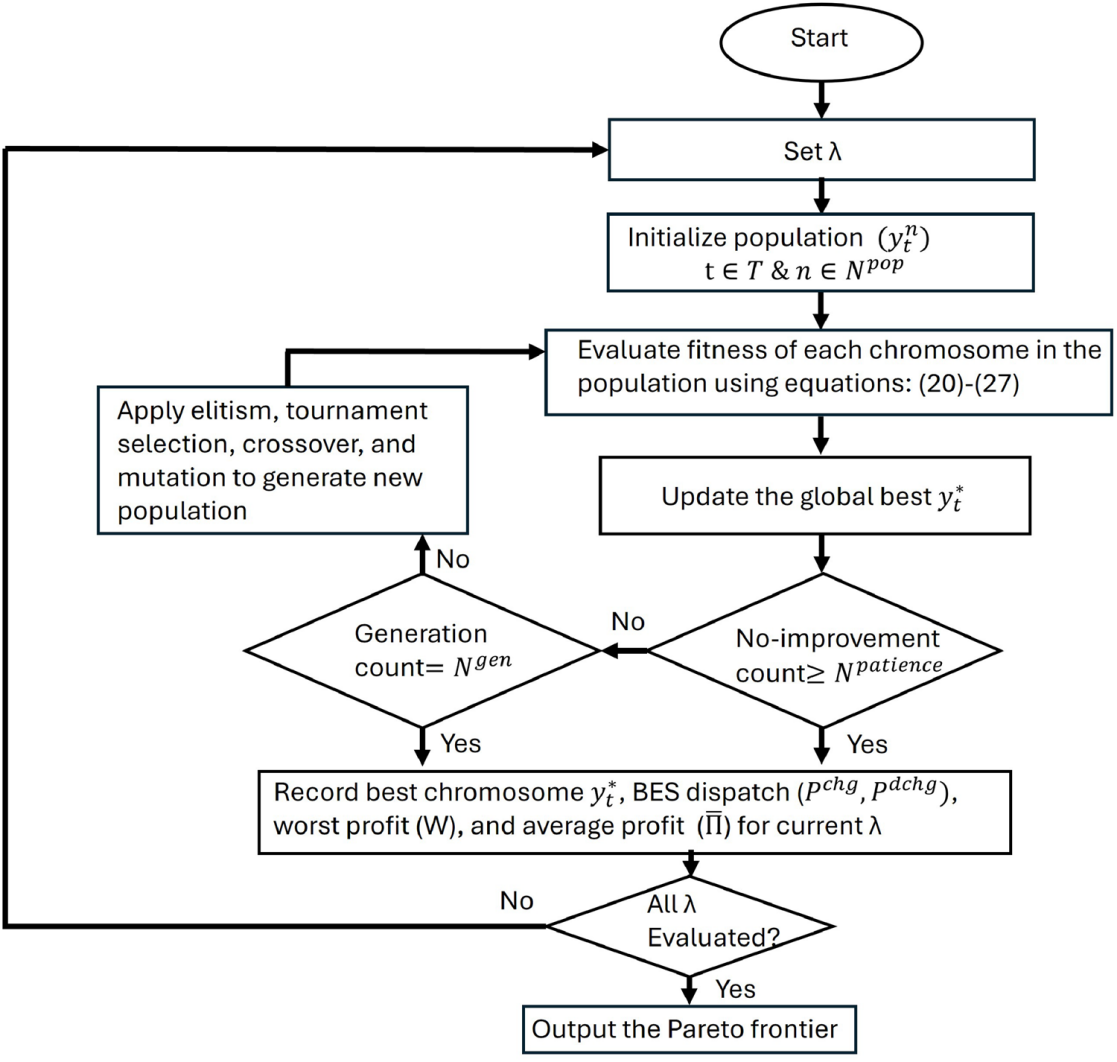
Flowchart of the proposed hybrid GA-LP day-ahead bidding strategy framework.

Figure 6 shows the main steps of the proposed GA-LP framework. The algorithm begins by initializing a population of candidate commitment schedules $\boldsymbol{y}$. Each candidate schedule is then evaluated using the coupled LP formulation discussed in the following subsection. The candidate schedule with the highest fitness value is tracked as the global best. A new population is then generated through tournament selection, crossover, mutation, and Elitism. The process continues until either of the stopping conditions is satisfied. It must be noted that GA is a heuristic optimization method. Therefore, the proposed framework does not guarantee convergence to a globally optimal solution. Nevertheless, convergence reliability was evaluated using multiple independent runs, as discussed in the results section.

### Coupled LP formulation

The second layer solves a coupled LP to determine the optimal BES dispatch schedule ($P_t^{\textrm{chg}}, P_t^{\textrm{dchg}}, SOC_t$), which is held fixed across all scenarios. The only variable that varies across scenarios is the grid exchange power ($P_t^{G,s}$). The coupled LP objective function is expressed in Eq. (18)

18 $$\begin{aligned} \begin{aligned} \max _{P_t^{\textrm{chg}},\,P_t^{\textrm{dchg}},\,P_t^{G}} \quad&\lambda \min _{s \in S} \Pi ^{s}(y) + (1-\lambda )\frac{1}{N_s}\sum _{s \in S}\Pi ^{s}(y) \end{aligned} \end{aligned}$$

The objective function includes two terms: the first represents the worst-case profit, while the second evaluates the average profit across all scenarios. The robustness weight $\lambda \in [0,1]$ controls the trade-off between robustness and profitability. Solving the optimization problem across various values of *λ* yields a Pareto frontier that supports operational decision-making. The profit under scenario *s* is defined as:

19 $$\begin{aligned} \begin{aligned} \Pi ^{s}(y)= \sum _{t \in T} \pi ^s_t P^{G,s}_t \Delta t \end{aligned} \end{aligned}$$

where $P_t^{G,s}$ is positive when power is exported to the grid and negative when power is imported. This scenario-dependent grid exchange captures the residual effect of PV generation uncertainty on the net power exchange. It should be noted that imbalance settlement charges are not modeled as penalty terms in the objective function. They are discussed as a motivation for the non-anticipative BES scheduling structure. Including imbalance charges would require an additional real-time settlement model and corresponding imbalance price data, which are beyond the scope of the present day-ahead scheduling framework. The non-anticipative structure adopted in this work therefore provides a practical approach to limiting reliance on scenario-dependent BES adjustments, which may otherwise increase imbalance exposure in real-time operation.

### Worst-case profit (robustness term)

The robustness term in (18) involves a *\min* operator, which leads to a non-smooth objective and cannot be directly handled by standard LP solvers. To linearize this term, an auxiliary variable *W* is introduced such that:

20 $$\begin{aligned} \begin{aligned} W \le \Pi ^{s}(y) \qquad \forall s \in S \end{aligned} \end{aligned}$$

By maximizing *W*, the optimizer pushes it to the minimum profit across all scenarios. At optimality:

21 $$\begin{aligned} \begin{aligned} W^* = \min _{s \in S} \Pi ^{s}(y) \end{aligned} \end{aligned}$$

The objective function can then be reformulated as:

22 $$\begin{aligned} \begin{aligned} \max _{P_t^{\textrm{chg}},\,P_t^{\textrm{dchg}},\,P_t^{G},W} \quad&\lambda W + (1-\lambda )\frac{1}{N_s}\sum _{s \in S}\Pi ^{s}(y) \end{aligned} \end{aligned}$$

### Average profit term

The average profit across all scenarios for a given BES commitment and dispatch schedule is computed by the second term of Eq. (22). The objective function is subject to the following physical and operational constraints:

23 $$\begin{aligned} \begin{aligned} P_t^{G,s} = P_t^{PV,s} + P_t^{\textrm{dchg}} - P_t^{\textrm{chg}} \qquad \forall t \in T, \forall s \in S \end{aligned} \end{aligned}$$

24 $$\begin{aligned} \begin{aligned} 0 \le P_t^{\textrm{dchg}} \le P^R u_t \qquad \forall t \in T \end{aligned} \end{aligned}$$

25 $$\begin{aligned} \begin{aligned} 0 \le P_t^{\textrm{chg}} \le P^R v_t \qquad \forall t \in T \end{aligned} \end{aligned}$$

where $u_t$ and $v_t$ are parameters derived from the GA decision variable $y_t$, which is fixed in the LP layer and defined as:

26 $$\begin{aligned} u_t = {\left\{ \begin{array}{ll} 1 & \text {if } y_t = 1 \\ 0 & \text {otherwise} \end{array}\right. } \qquad v_t = {\left\{ \begin{array}{ll} 1 & \text {if } y_t = -1, \\ 0 & \text {otherwise} \end{array}\right. } \qquad \forall t \in T \end{aligned}$$

The energy stored in the battery for each time interval is given by:

27 $$\begin{aligned} \begin{aligned} E_{t+1} = E_t + \eta ^{\textrm{chg}} P_t^{\textrm{chg}} \Delta t - \frac{P_t^{\textrm{dchg}} \Delta t}{\eta ^{\textrm{dchg}}}, \qquad \forall t \in T \end{aligned} \end{aligned}$$

28 $$\begin{aligned} \begin{aligned} (1-DOD)E^{\text {R}} \le E_t \le E^{\text {R}}, \qquad \forall t \in T \end{aligned} \end{aligned}$$

29 $$\begin{aligned} \begin{aligned} E_{24} = E_0 \end{aligned} \end{aligned}$$

Equation (23) maintains power balance condition at each time step. The charging and discharging power limits are constrained by (24) and (25). Since $y_t$ *∈* *[-1, 0, +1]*, mutual exclusivity between charging and discharging is inherently guaranteed through $u_t$ and $v_t$, eliminating the need for additional binary variables. The hourly SOC is calculated using (27). The BES energy limits (28–29) include the maximum depth-of-discharge constraint, which prevents excessive depletion of the battery and provides a basic operational protection related to battery health. However, cycle-related battery degradation costs are not explicitly included in the present formulation. This simplifying assumption keeps the LP layer linear and allows the analysis to focus on the proposed uncertainty-aware day-ahead bidding framework and the profitability–robustness trade-off. Incorporating detailed degradation models is left for future work.

## Case study

### Case study setup

The case study is conducted using real-world PV generation and electricity price data. The PV generation data are obtained from the National Renewable Energy Laboratory (NREL) solar power dataset^27^, while day-ahead electricity price data are sourced from the PJM Interconnection data viewer for locational marginal prices (LMP)^24^. Table 2 summarizes the main characteristics of the datasets used in this study. For PV output power scenario generation, historical data from June 1 to August 30, 2006 are used, excluding the target day (July 16, 2006), which serves as the day-ahead scheduling horizon.

**Table 2:** Dataset description and scenario generation setup.

Parameter	Value
Data type	Real-world dataset
Location (Lat, Long)	*37.55∘*, *-89.45∘*
Weather year	2006
PV technology	Single axis tracking, utility scale PV
Installed capacity (MW)	106 MW
Historical period	June 1 – August 30
Target day	July 16
Excluded from dataset	Yes
Number of scenarios ($N_s$)	100

The jump diffusion model and the Gaussian copula method are used to generate the electricity price and PV generation scenarios, respectively, as described in the previous sections.

The BES characteristics are summarized in Table 3. The selected BES energy and power ratings are defined relative to the PV system capacity to ensure practical integration. In particular, the BES power rating of 100 MW corresponds to approximately 94 *%* of the PV plant peak output (*≈*106 MW). In addition, a 4-hour energy-to-power ratio is adopted, which is consistent with typical utility-scale battery storage configurations.

**Table 3:** Battery energy storage system (BES) parameters.

Parameter	Description	Value	Unit
$E^{\text {R}}$	Rated BES energy capacity	400	MWh
$\textrm{DOD}$	Depth of discharge	0.80	–
$P^{\text {R}}$	Maximum charging/discharging power	100	MW
$\eta ^{\textrm{chg}}$	Charging efficiency	0.90	–
$\eta ^{\textrm{dchg}}$	Discharging efficiency	0.90	–
$\textrm{E}_0$/ $\textrm{E}_{24}$	Initial/terminal stored energy $(= 0.5E^{\text {R}})$	200	MWh

### Optimization setup

The GA layer determines the BES commitment schedule using an evolutionary search process. The GA parameter settings are given in Table 4. The initial population is randomly generated by sampling each gene independently from ${-1,0,1}$. A fixed random seed of 42 is used to ensure reproducibility. For each candidate schedule, the fitness value is computed by solving the coupled LP formulation described in the previous subsection. Tournament selection is employed, followed by single-point crossover and mutation to evolve the population over successive generations. Elitism is applied by carrying the best candidate schedule unchanged into the next generation. To reduce computation time, an early stopping criterion is applied when no improvement in the best fitness value is observed for $N_{pop}$ generations.

**Table 4:** Genetic algorithm (GA) parameter settings.

Parameter	Symbol	Value
Population size	$N_{\textrm{pop}}$	30
Maximum generations	$N_{\textrm{gen}}$	100
Early-stop patience	$N_{\textrm{patience}}$	15
Crossover probability	$p_c$	0.80
Mutation probability	$p_m$	0.05
Tournament size	$k_{\textrm{tourn}}$	3
Elitism	–	1 individual

The LP layer determines the scenarios-shared optimal BES dispatch schedule for a given commitment schedule $\boldsymbol{y}$, enforcing a non-anticipative decision structure and providing an exact evaluation of the fitness function.This resulted in a total of $n_{\textrm{var}} = 3T + TN_s + 1$ decision variables, which for *T = 24*, $N_s = 100$ yielding 2,473 decision variables.

### Benchmark methods

To evaluate the performance of the proposed framework, the same case study is solved using a deterministic MILP approach, a naive rule-based approach, and a classical robust optimization (RO) benchmark based on a box uncertainty set. The naive approach commits the BES to discharge during the (*N=2*) highest-price forecast hours and charge during the (*N=2*) lowest-price forecast hours, with the power magnitudes optimized by an LP on the day-ahead forecast. The deterministic MILP and naive approaches rely on day-ahead forecasts of PV generation and electricity price without accounting for uncertainty.

The box RO maximizes the worst-case profit over a continuous uncertainty set whose price and PV bounds at each hour are set to the minimum and maximum values observed across the 100 generated ^20^. The box RO enforces the same non-anticipative shared dispatch structure as the proposed framework, and uses the same binary commitment formulation as the deterministic MILP benchmark.

30 $$\begin{aligned} \max _{\textbf{y},\, P_t^{\textrm{chg}},\, P_t^{\textrm{dchg}}} \quad \min _{\begin{array}{c} \pi _t \in [\pi _t^L, \pi _t^U] \\ P_t^{PV} \in [P_t^{PV,L}, P_t^{PV,U}] \end{array}} \quad \sum _{t=1}^{T} \left( P_t^{PV} + P_t^{\textrm{dchg}} - P_t^{\textrm{chg}} \right) \pi _t \, \Delta t \end{aligned}$$

31 $$\begin{aligned} \pi _t^L = \min _{s}\, \pi _t^s, \qquad \pi _t^U = \max _{s}\, \pi _t^s, \qquad \forall \, t \in T \end{aligned}$$

32 $$\begin{aligned} P_t^{PV,L} = \min _{s}\, P_t^{PV,s}, \qquad P_t^{PV,U} = \max _{s}\, P_t^{PV,s}, \qquad \forall \, t \in T \end{aligned}$$

All three benchmarks enforce a fixed day-ahead dispatch plan held constant across all 100 scenarios, ensuring a consistent evaluation basis with the proposed framework.

## Results and discussion

**Fig. 7:**
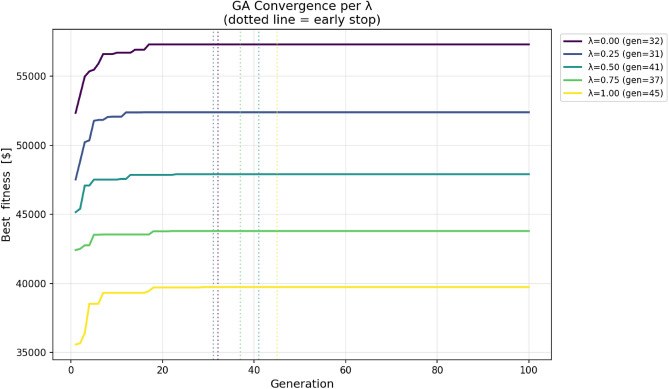
GA convergence curves for different *λ*.

The GA convergence curves for different values of the robustness weight *λ* are shown in Fig. 7. The results show that the GA converges to a solution within 32 to 45 generations, well before the maximum generation limit of 100. The highest number of generation is observed when *(λ =1)*, indicating the added difficulty of determining a commitment schedule that consistently protects against all 100 scenarios simultaneously.

**Fig. 8:**
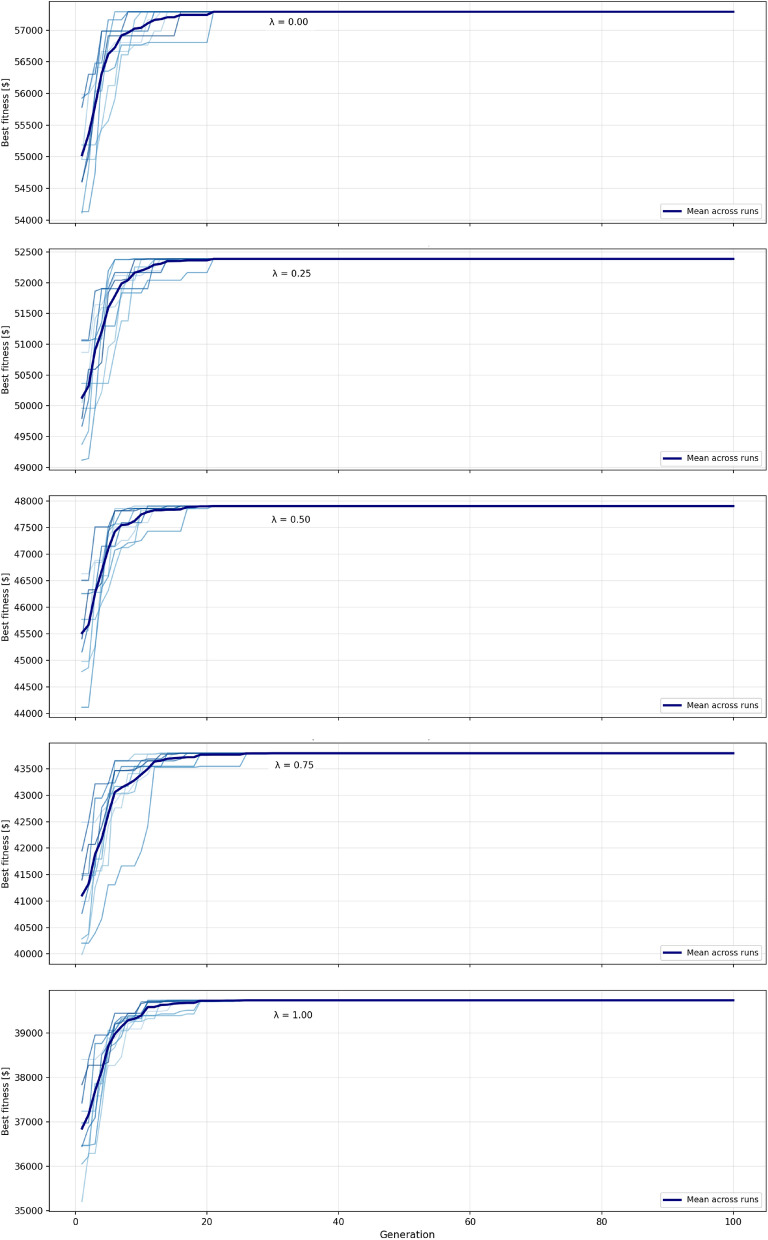
GA convergence curves across multiple independent runs with different initial populations for each robustness weight *λ*.

To evaluate the convergence reliability of the GA, 10 independent runs were conducted for each *λ* value using different initial random populations while keeping the scenario set fixed. The convergence curves for all runs and all *λ* values are shown in Fig. 8. All 10 runs converged to the same optimal objective value across all five *λ* values, with a standard deviation of zero in every case. As observed in the figure, while different initial populations explore different regions of the search space during the early generations, all runs consistently converge to the same solution within 20–45 generations, well before the maximum generation limit of 100. The number of generations required for convergence increases slightly with *λ*, from approximately 27 generations at *λ =0* to approximately 38 generations at *λ =1*, reflecting the increasing difficulty of optimizing against all scenarios simultaneously as the robustness weight increases. These results provide evidence that the selected GA parameters yield consistent solutions and do not exhibit sensitivity to the initial population.

**Fig. 9:**
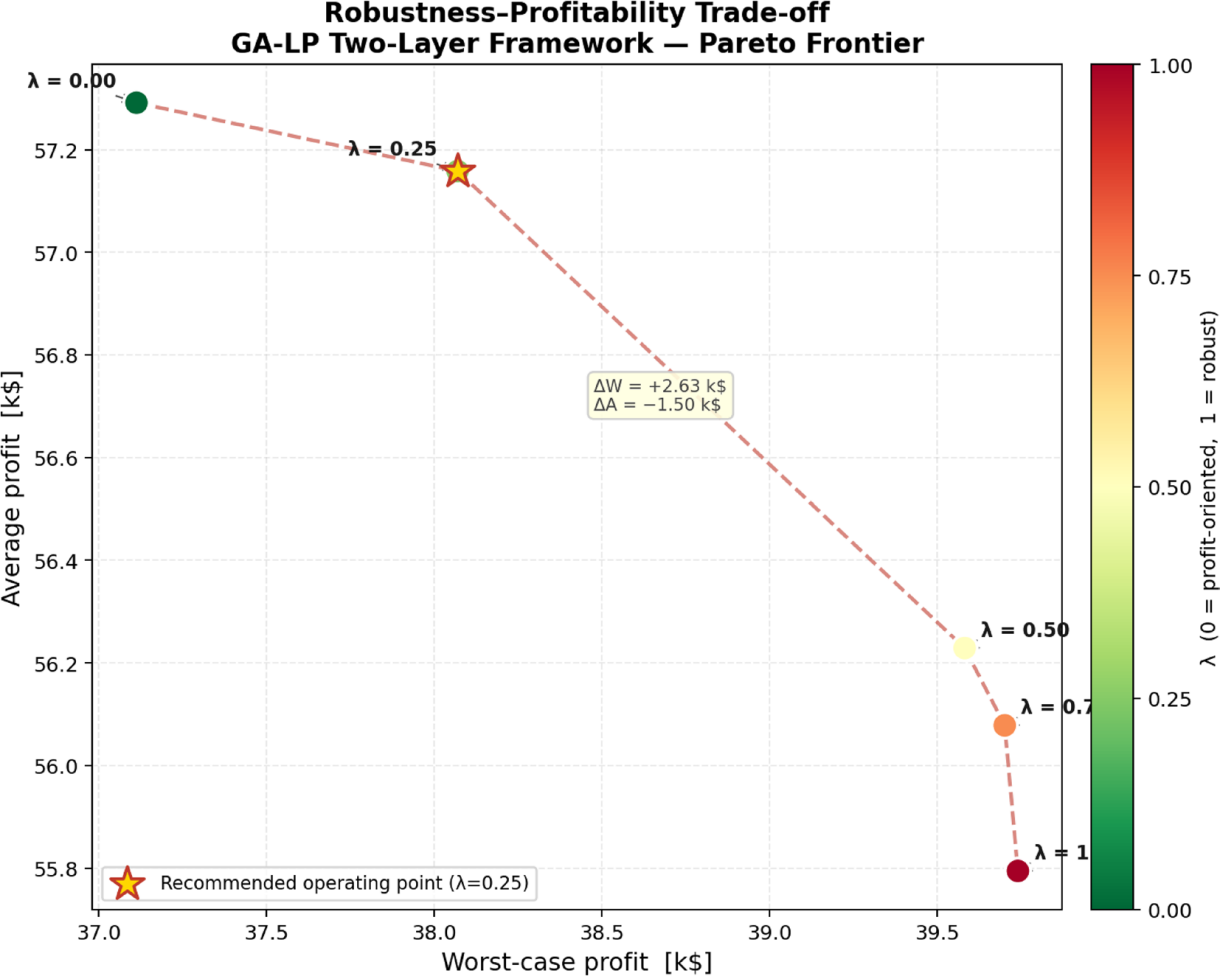
Obtained Pareto frontier.

The trade-off between robustness and profitability is illustrated by the Pareto frontier shown in Fig. 9. As *λ* increases, the objective function shifts from profitability to robustness, reflected in a reduction in average profit and an increase in the worst-case profit. Table 5 presents the detailed economic results of the case study. The parameters *Δ W* and $$\Delta \bar{\Pi }$$ represent the gain in worst-case profit and the loss in average profit with respect to the preceding value of *λ*. Moving from the fully profit-oriented solution (*λ =0*) to the fully robust solution (*λ =1*), the worst-case daily profit increases by $ 2,628 (+7.1%) while the average daily profit decreases by $1,497 (*-2.6%*). The efficiency ratio, defined as the ratio of *Δ W* to $$\Delta \bar{\Pi }$$, represents the worst-case profit gained per dollar of average profit sacrificed. At the first transition ($$\lambda = 0 \rightarrow 0.25$$), the ratio reaches its maximum value of 7.19, indicating that a small shift toward robustness yields large worst-case improvements at a low average profit cost. Beyond *λ = 0.50*, the efficiency ratio falls below unity (0.79 and 0.14 at *λ = 0.75* and 1.00, respectively), indicating that further robustness investment yields diminishing returns. Based on this analysis, *λ = 0.25* is identified as the recommended operating point, as it captures *36%* of the total achievable worst-case improvement at only *9%* of the total average profit cost.

**Table 5:** Cost-benefit efficiency analysis of the Pareto frontier.

* λ *	*W*($)	$$\bar{\Pi }$$($)	* Δ W *($)	$$\Delta \bar{\Pi }$$($)	Efficiency ratio	Remark
0.00	37 112	57 291	—	—	—	Baseline
0.25	38 070	57 158	+957	−133	7.19	Most efficient
0.50	39 582	56 229	+1 512	−929	1.63	Moderate
0.75	39 701	56 078	+119	−151	0.79	Diminishing
1.00	39 740	55 795	+39	−284	0.14	Fully robust
Total ($\lambda = 0 \rightarrow 1$)			+2 628	−1 497	—	+7.1%*W*, −2.6%$\bar{\Pi }$

Table 6 shows a detailed performance comparison between the proposed framework with the three selected benchmark methods. The deterministic MILP achieves an average profit of $57,274 and a worst-case profit of $37,231. The proposed framework at *λ = 0* produces nearly identical results ($$\bar{\Pi } = \$57{,}291$$, $$W = \$37{,}113$$), differing by less than $200 on both metrics. This close agreement shows that when robustness is not prioritized (i.e., *λ = 0*), the proposed framework returns a solution close to the deterministic solution. This indicates that the uncertainty-aware formulation does not introduce systematic bias or conservatism.

Based on the Pareto frontier analysis, *λ = 0.25* is identified as the recommended operating point. Compared to the deterministic benchmark, this solution improves the worst-case profit by $839 (*+2.3%*) while reducing the average profit by only $116 (*-0.2%*),indicating a meaningful gain in worst-case protection at a negligible average profit cost. The $$W/\bar{\Pi }$$ ratio also improves from *65.0%* for the deterministic method to *66.6%* at *λ = 0.25*, reflecting more consistent performance across all uncertainty scenarios. At the fully robust solution (*λ = 1.00*), the worst-case profit reaches $39,740, representing a $2,509 improvement (*+6.7%*) over the deterministic benchmark, with a $$W/\bar{\Pi }$$ ratio of *71.2%* compared to *65.0%* for the deterministic approach. However, this comes at a cost of $1,479 in average profit (*-2.6%*), which may be acceptable for operators with a strong preference for downside risk mitigation.

The naive rule-based approach achieves the lowest average profit and worst-case profit among all methods considered. Its $$W/\bar{\Pi }$$ ratio of *60.0%* is the lowest of all methods, reflecting the high sensitivity of the naive schedule to unfavorable scenario realizations. Compared to the recommended proposed framework solution at *λ = 0.25*, the naive approach yields a worst-case profit that is $8,423 lower (*-22.1%*) and an average profit that is $7,775 lower (*-13.6%*). This highlights the importance of considering uncertainty in the day-ahead bidding strategies to reduce the risk of financial losses during real time operation.

The classical robust optimization benchmark yields a worst-case profit of $29,278 and an average profit of $47,032, with a $$W/\bar{\Pi }$$ ratio of 62.3%. Despite being an uncertainty-aware method, the box RO performs worse than the deterministic MILP on both metrics, and worse than all solutions on the proposed framework’s Pareto frontier. This outcome reflects the well-documented over-conservatism of classical box robust optimization^17^, which optimizes against a continuous uncertainty set that includes synthetic worst-case combinations that are unlikely to occur in practice. The proposed framework, by contrast, optimizes directly against the discrete scenario set that captures realistic joint realizations of PV generation and electricity price uncertainty. At the recommended operating point of *λ =0.25*, the proposed framework improves worst-case profit by $8,792 (+30.0%) and average profit by $10,126 (+21.5%) over the box RO benchmark. Moreover, every solution on the Pareto frontier simultaneously dominates the box RO in both worst-case and average profit, demonstrating that the scenario-based robustness formulation provides strictly superior practical performance to classical box robust optimization for this problem.

**Table 6:** Performance comparison of the proposed hybrid GA-LP framework against deterministic, naive rule-based, and robust optimization benchmarks.

Method	* λ *	*W*($)	* Π *($)	* W/Π *(%)	D	I	C
Deterministic MILP	–	37,231	57,274	65.0	18	0	6
Naive rule-based	–	29,647	49,384	60.0	2	20	2
Robust optimization	–	29,278	47,032	62.3	8	0	16
GA–LP Framework (proposed)	0.00	37,113	57,291	64.8	7	8	9
GA–LP Framework (proposed)	0.25$^{*}$	38,070	57,158	66.6	10	3	11
GA–LP Framework (proposed)	0.50	39,582	56,229	70.4	10	3	11
GA–LP Framework (proposed)	0.75	39,701	56,078	70.8	9	2	13
GA–LP Framework (proposed)	1.00	39,740	55,795	71.2	10	1	13

The asterisk indicates the recommended operating point on the Pareto frontier, corresponding to the highest incremental cost-benefit efficiency ratio.

**Fig. 10:**
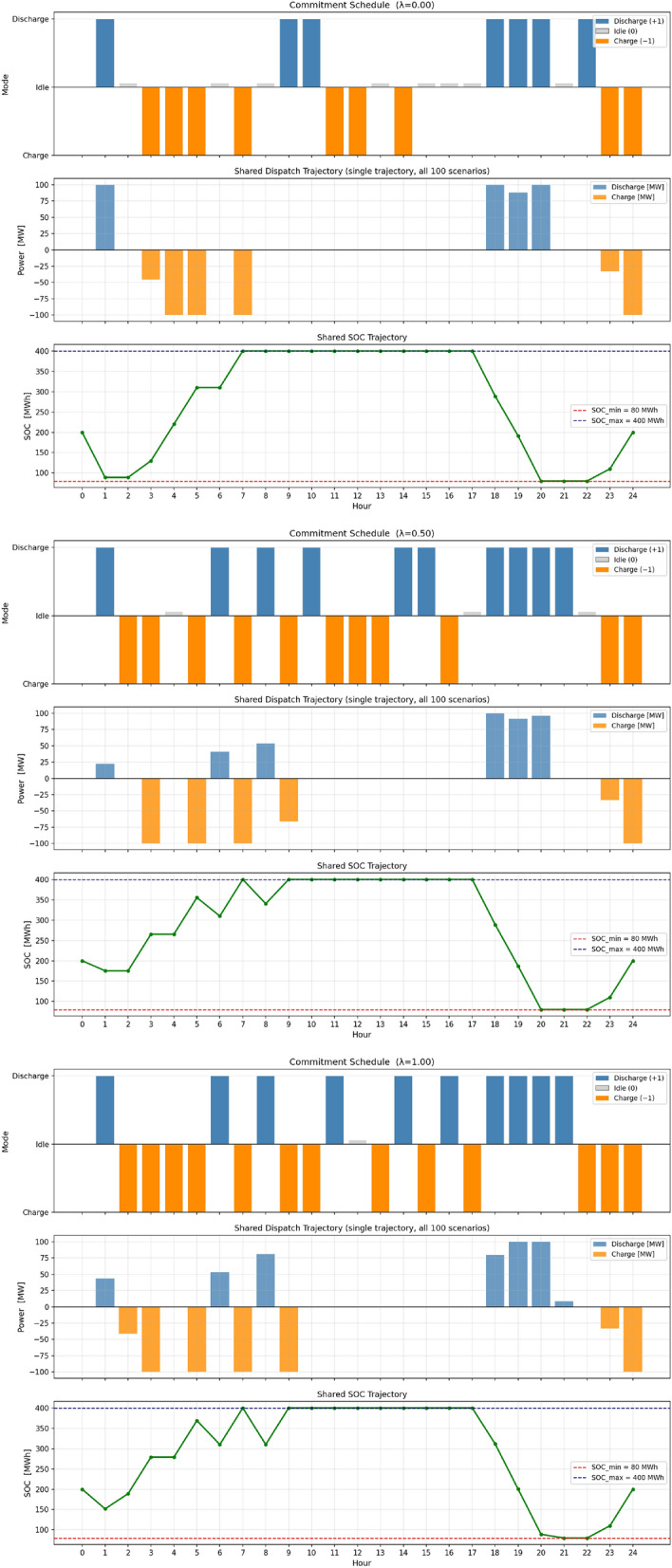
BES schedules under different *λ* values.

The optimal commitment and dispatch schedules for *λ =0* ,0.5, and 1.00 are shown in Fig. 10, the profit-oriented schedule commits to 7 discharge hours, 9 charge hours, and 8 idle hours. As *λ* increases to 1.00, idle hours collapse to just 1 while charge hours increase to 13. This reveals a counterintuitive but physically meaningful result: the robust schedule is more active, not less. The GA finds that robustness is best achieved by maintaining a fuller battery state through more aggressive charging commitments, thereby preserving discharge capacity for the scenarios in which it is most needed. The shared SOC trajectories confirm that all schedules satisfy the physical constraints – SOC remains within [80, 400] MWh throughout the day and returns to the initial value of 200 MWh at hour 24.

## Data Availability

Electricity price data were obtained from the PJM Interconnection data viewer (https://dataviewer.pjm.com/) and PV generation data were obtained from the NREL solar power dataset (https://www.nrel.gov/grid/solar-power-data).
